# Substrate curvature influences cytoskeletal rearrangement and modulates macrophage phenotype

**DOI:** 10.3389/fimmu.2024.1478464

**Published:** 2025-01-06

**Authors:** Austin Sovar, Matthew D. Patrick, Ramkumar T. Annamalai

**Affiliations:** ^1^ Department of Biomedical Engineering, University of Kentucky, Lexington, KY, United States; ^2^ Department of Biomedical Engineering, University at Buffalo, Buffalo, NY, United States

**Keywords:** microgel, gelatin, immunomodulation, macrophages, curvature, inflammation, F-actin, MRTF-A

## Abstract

**Introduction:**

Inflammation is a vital immune response, tightly orchestrated through both biochemical and biophysical cues. Dysregulated inflammation contributes to chronic diseases, highlighting the need for novel therapies that modulate immune responses with minimal side effects. While several biochemical pathways of inflammation are well understood, the influence of physical properties such as substrate curvature on immune cell behavior remains underexplored. This study investigates how substrate curvature impacts macrophage cytoskeletal dynamics, gene expression, and immunophenotype through mechanosensitive pathways.

**Methods:**

Gelatin-based microgels with tunable surface curvatures were fabricated via water-in-oil emulsification and crosslinked with genipin. Microgels were sorted into three size ranges, yielding high (40-50 µm), intermediate (150-250 µm), and low (350-400 µm) curvature profiles. Macrophages were seeded onto these microgels, and cytoskeletal dynamics were examined using confocal microscopy, SEM, and actin-specific staining. Gene expression of pro- and anti-inflammatory markers was quantified using qPCR. The role of actin polymerization was assessed using Latrunculin-A (Lat-A) treatment.

**Results:**

Macrophages adhered effectively to both high- and low-curvature microgels, displaying curvature-dependent morphological changes. Confocal imaging revealed that macrophages on low-curvature microgels exhibited significantly higher F-actin density than those on high-curvature microgels. Correspondingly, qPCR analysis showed upregulation of pro-inflammatory markers (e.g., Tnf, Nos2) in high-curvature conditions, while anti-inflammatory markers (e.g., Arg1) were elevated in low-curvature conditions. Lat-A treatment reduced F-actin density and modulated gene expression patterns, confirming the cytoskeletal regulation of macrophage phenotype.

**Discussion:**

These findings demonstrate that substrate curvature influences macrophage behavior by modulating cytoskeletal dynamics and associated immunophenotypic markers through actin-mediated transcriptional pathways. By controlling curvature, therapeutic biomaterials may direct immune responses, offering a new avenue for treating inflammatory diseases. This mechanobiological approach presents a promising strategy for precision immunomodulation in regenerative medicine.

## 1 Introduction

Inflammation is a critical component of the immune system, serving as the body’s first line of defense against foreign threats or injurious stimuli. It plays a vital role in fighting infections, clearing damaged cells, and initiating tissue repair. When effectively regulated, the inflammatory response resolves threats promptly and transitions into a healing state. However, when dysregulated, the inflammation can become prolonged, impeding repair, exacerbating tissue damage and promoting fibrosis. This chronic inflammatory response is a fundamental aspect of several degradative and chronic conditions, including diabetes, cardiovascular disease, arthritis, and various organ-specific diseases (1–3) that affect populations globally. The World Health Organization (WHO) recognizes chronic inflammation-related diseases as a significant threat to global health. For instance, in the US alone, cardiovascular disease accounts for 800,000 deaths annually, and around 30.3 million people suffer from diabetes (1). These conditions often share a common underlying mechanism involving dysregulated inflammatory and immune responses, highlighting the urgent clinical need for therapies to mitigate these harmful processes.

Macrophages, versatile innate immune cells, play a pivotal role in the initiation, progression, and resolution of inflammation (4). These cells exhibit remarkable plasticity, dynamically adapting their phenotypes to environmental cues. The M1 macrophage phenotype, or “classically activated” state, is biochemically induced by microbial components like lipopolysaccharides (LPS) or pro-inflammatory cytokines such as IFN-γ. M1 macrophages are instrumental in pathogen clearance and the promotion of inflammation, producing cytokines such as IL-6, IL-12, and TNF-α while relying on glycolytic metabolism. These cells are characterized by markers like iNOS and inflammatory chemokines. In contrast, M2 macrophages, or “alternatively activated” macrophages, are stimulated by anti-inflammatory cytokines like IL-4 and IL-13. These cells are associated with tissue repair and the resolution of inflammation, secreting cytokines such as IL-10 and TGF-β and employing oxidative phosphorylation and fatty acid metabolism. These cells are characterized by markers like arginase-1, CD163, and CD206. Notably, macrophages operate along a functional spectrum, transitioning between phenotypes based on their microenvironment, enabling a tailored response to diverse biological needs (5–7).

While both biochemical and biophysical cues are known to influence macrophage polarization, the mechanisms underlying these effects remain poorly understood (8). A deeper understanding of these mechanisms would enable the harnessing of macrophage plasticity to modulate their immunophenotype, potentially resolving dysregulated immune responses. Numerous studies have demonstrated successful approaches to modulating macrophage immunophenotypes (9–13). Systemic application of biochemical agents, such as JAK inhibitors, which inhibit the activity of Janus kinases (14) and proinflammatory cytokines such as IL-1β and TNFα (15, 16), have shown efficacy in alleviating chronic inflammation. Similarly, humanized monoclonal antibodies targeting IL-6R to antagonize IL-6 binding and hinder inflammatory responses are currently utilized in treating several inflammatory conditions (17). However, these biochemical factors often lack target specificity and can increase susceptibility to infection and systemic toxicity (18). This underscores the need for targeted and localized approaches that can modulate the immune response without relying on soluble factors.

Biophysical cues such as substrate stiffness (19), viscoelasticity (20), geometry (21), and spatial patterns (22) have shown the potential to address this challenge (23). Cells primarily recognize these physical cues through integrin-mediated mechanotransduction pathways, which convert mechanical forces into biochemical signals, subsequently altering cytoskeletal dynamics and phenotype (24). Macrophages, in particular, respond to these physical cues through actin-cytoskeletal reorganization, nuclear deformation, and subsequent gene expression (25).

**Figure 1:**
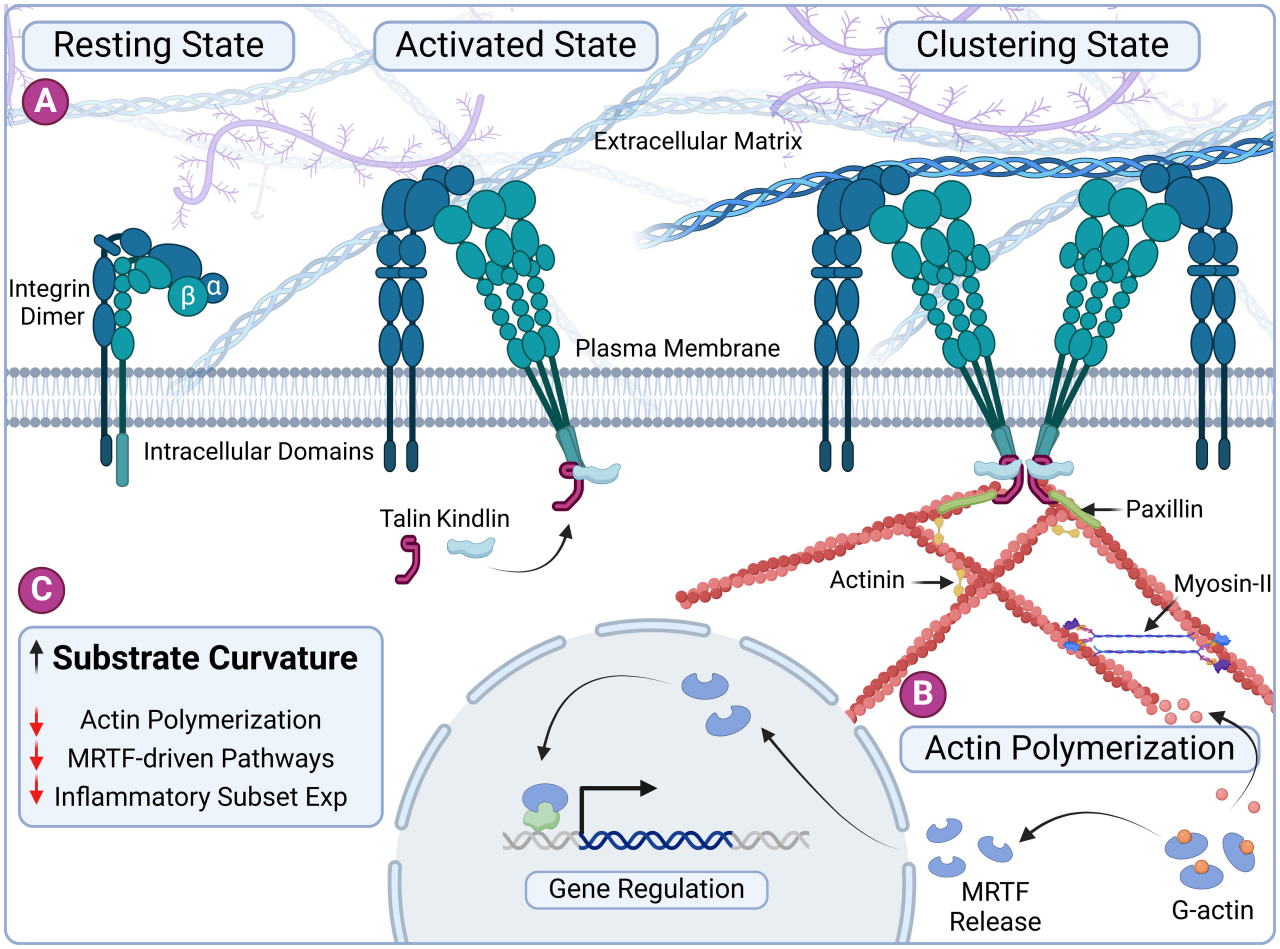
Substrate curvature influences macrophage phenotype through cytoskeletal dynamics. **(A)** Schematic representation of integrin-based nascent adhesion formation. The process begins with the activation of integrins by talin and kindlin, leading to nucleation of ligand-bound integrin clusters. Adhesome proteins such as vinculin and paxillin are subsequently recruited to these adhesion sites, stabilizing the adhesion complexes. **(B)** The polymerization of G-actin into F-actin releases the transcription factor MRTF. MRTF translocates to the nucleus, where it regulates the expression of inflammatory genes, linking cytoskeletal dynamics to immunomodulatory functions. **(C)** Overview of our central hypothesis: Increased substrate curvature imposes a bending energy penalty and reduces actin polymerization, ultimately influencing macrophage immunophenotype through curvature-induced cytoskeletal remodeling. Created in BioRender. Annamalai RT (2024) https://BioRender.com/s88l335.

Here, we describe a straightforward approach to modulate macrophage responses using simple gelatin-based microgels with tunable properties, including size and curvatures, capable of inducing a localized immunomodulatory effect. We hypothesize that curvature influences macrophage behavior through actin polymerization, primarily via the myocardin-related transcription factor A (MRTF-A) pathway—a mechanosensitive regulator of gene expression (**Figure 1**). To test the hypothesis, we fabricated tunable spherical microgels with varying curvatures and characterized their biophysical properties. Our findings indicate that macrophages readily attach to the surface of these microgels regardless of their curvature. However, the curvature of the substrate significantly influences F-actin polymerization, leading to changes in macrophage phenotype. This effect was partially reversed when treated with selective actin inhibitor Latrunculin A. These results demonstrate the influence of substrate curvature on macrophage cytoskeletal dynamics and resulting immunophenotype. Consequently, this simple approach has the potential to be utilized as a localized immunomodulatory treatment for inflammatory diseases.

## 2 Methods

### 2.1 Microgel fabrication

Microgels were fabricated as previously described (26). Briefly, a 6% gelatin (type A, 300 bloom, Sigma) in deionized (DI) water stock solution was created. This solution was dispensed dropwise into a polydimethylsiloxane (PDMS, viscosity = 100 cS, Clearco Products Co., Inc.) bath heated to 37°C. The mixture was emulsified with a double impeller at either 500 or 1000 rpm to achieve the desired size range. After 5 minutes of emulsification at 37°C, the mixture was cooled using an ice bath and stirred for 30 minutes. The microgels were then separated through centrifuging at 185 g for 5 minutes. The supernatant was discarded, and the resulting microgel pellet was washed thrice with Dulbecco phosphate buffer solution (PBS, Invitrogen) supplemented with 1% TWEEN 20 (Sigma). The microgels were then crosslinked with 1 wt% genipin in PBS for 6 or 48 hours to achieve different crosslinking densities. After cross-linking, the excess genipin was removed by washing with 100% ethanol and stored in 100% ethanol at 4-8°C until further use.

To obtain microgels of specified size and curvature, they were washed and swollen in PBS, sonicated for 5 minutes in an ice bath, and sorted using nylon mesh filters. For our studies, 40-50 µm (κ = 0.045 µm-1), 150-250 μm, and 350-400 μm (κ = 0.005 µm^-1^) nylon meshes were used. This procedure was repeated twice to remove aggregates thoroughly.

### 2.2 Swelling and polymer density

Microgel swelling and polymer density were characterized using microgels within the size range of 150-250 μm crosslinked for 6 hours (low crosslinking) and 48 hours (high crosslinking). The mass and volume swelling ratio were determined and used to find the polymer densities. The swelling ratios were calculated through quantitative measurements from hydrated and dehydrated microgels. For hydration, the microgels were swollen in 10 mM PBS before measurements. Dehydration was achieved through flash freezing in liquid nitrogen followed by lyophilization. The polymer density was calculated using the following equation:


$$\frac{V_{h}}{V_{d}}=1+\frac{\rho_{p}}{\rho_{pbs}}\left(\frac{m_{h}}{m_{d}}-1\right)$$

Where $V_{h}$ is the volume of hydrated spheres, $V_{d}$ is the volume of dry spheres, $\rho_{p}$ is the polymer density, $\rho_{pbs}$ is the density of microgels after swollen in PBS, $m_{h}$ is the mass of hydrated spheres, and $m_{d}$ is the mass of the dehydrated spheres. Bright-field images of the microgels were obtained and processed in ImageJ to measure the diameter and calculate the volume of spheres.

### 2.3 Compression testing

The mechanical properties of the microgel formulations were characterized and compared using compression testing. Briefly, cylindrical bulk gels were prepared in a 24-well flat bottom plate, crosslinked with 1 wt% genipin in PBS, washed with ethanol at specific time points to stop the reactions, and then rehydrated in 10 mM PBS for testing. Uniform disks of 5 mm in height were cut out using a 6 mm biopsy punch. The cylindrical samples were subjected to compression testing using a Universal Testing Machine (Instron). The samples were compressed at a strain rate of 10 mm/min, generating a force-displacement curve. The elastic modulus was then calculated from the linear region (between 5-15% strain region) of the resulting stress-strain curve.

### 2.4 Cell culture

A murine macrophage cell line IC-21 (ATCC) was cultured with RPMI-1640 Medium (ATCC) supplemented with 10% fetal bovine serum, anti-biotic and anti-mycotic supplements. Cells were expanded and maintained at 37°C in a CO_2_ incubator. Macrophages were grown to ~80% confluency then detached and used for experimental use.

For curvature studies, microgels (~3 mg/mL) of the desired curvature (40-50 µm for low curvature and 350-400 µm for high curvature) were seeded with ~3 million macrophages. To prepare the microgels for cell seeding, they were swollen with media in vented culture tubes (VWR), which were then replaced with macrophages suspended in growth media. The seeded microgels were kept in the incubator and gently mixed every 10 minutes for 1 hour to prevent aggregation. They were then washed with fresh media to remove unattached cells and transferred to a 35 mm bioinert µ-dishes (Ibidi) to prevent aggregation. For Latrunculin A (LatA) studies 100 nM of LatA was added to samples 24 hours before harvesting samples.

### 2.5 RNA isolation and quantitative gene expression

Total RNA was isolated from samples by first removing media from the suspension and adding 500 µL of TRIzol. The mixture was then vortexed vigorously, centrifuged at 10,000 g for 5 minutes, and the supernatant was transferred to a new 1.5 mL tube. Subsequently, 100 µL of chloroform was added, mixed vigorously, incubated at room temperature for 10 minutes, and then centrifuged at 16,000 g at 4°C for 10 minutes. The aqueous phase was transferred to a new 1.5 mL tube where 10 µg of glycogen and 250 µL of isopropanol were added. This mixture was then mixed, incubated at room temperature, and centrifuged at 12,000 g at 4°C for 10 minutes. The supernatant was discarded, and 500 µL of 75% ethanol was added to the remaining pellet, which was then vortexed and centrifuged at 7,500 g at 4°C for 10 minutes. After discarding the supernatant, the pellet was allowed to air dry. 10-20 µL of RNAse-free water was added to the dry pellet, and the solution was heated at 55°C for 15 minutes. The quality of isolated RNA was assessed through absorbance measurement using NanoDrop (ThermoFisher).

With isolated RNA samples, qPCR gene expression analysis was conducted. In each qPCR well, RNA samples were combined with RNAse free water, 2x reaction buffer, ROX, SuperScript, and TaqMan probe in a 10:50:2:2:31:5 ratio, respectively. Various TaqMan probes were employed to identify distinct gene expressions. The Ct values of *Gapdh* (Mm99999915_g1), *Actb* (Mm02619580_g1), and *Hsp90ab1* (Mm00833431_g1) were averaged and used as internal controls. *Arg1* (Mm00475988_m1) and *Igf1* (Mm00439560_m1) were used to characterize M2-prohealing genes. *Il-1b* (Mm00434228_m1), *Nos2* (Mm00440502_m1), and *Tnf* (Mm00443260_g1) were used to characterize M1-proinflammatory genes. Arpc2 (Mm01254383_m1), Cfl1 (Mm03057591_g1), and Tln1 (Mm00456997_m1) were used to quantify changes in cytoskeletal proteins expressions. Expression of genes seeded on high and low curvature microgels for 1 day were used as the baseline for each respective group. For normalization and comparison, gene expression levels of cells seeded on high- and low-curvature microgels for 24 hours were set as the baseline for their respective groups. Changes in gene expression were determined using the -ΔΔCt method, with expression levels normalized to the average Ct values of the internal control genes. Results were presented as fold changes relative to the baseline. All qPCR analyses were performed using a QuantStudio 3 Real-Time PCR System (Thermo Fisher Scientific). Data were presented as mean ± standard deviation for each gene and condition, illustrating how microgel curvature modulates macrophage gene expression profiles.

### 2.6 Electron microscopy

Samples intended for electron microscopy were fixed with glutaraldehyde. The fixed samples were dehydrated in 100% ethanol, flash-frozen in liquid nitrogen, and lyophilized. The Leica ACE 600 was used to sputter coat the lyophilized samples with a 5 nm-thick layer of platinum. Images were taken using the FEI Quanta 250 environmental scanning electron microscope (SEM) at 2 kV.

### 2.7 Immunofluorescent staining and confocal imaging

Samples intended for immunofluorescent staining were fixed with Z-fix (Anatech). Prior to staining, fixed samples were permeabilized with 0.1% Triton X in PBS for 3-5 minutes and then washed with 10 mM PBS. The samples were stained with Phalloidin-FITC (Molecular Probes) and DAPI (Molecular Probes) to target the F-actin cytoskeleton and DNA in the nucleus, respectively. The staining solution consisted of Phalloidin at 1:40 and DAPI at 1:1000 ratio with 1% BSA in PBS. Samples were stained for 30 minutes in the dark and washed twice with PBS.

Z-stack confocal images were acquired using a Nikon A1R confocal microscope and processed using ImageJ software. F-actin density was quantified by demarking macrophage boundaries and measuring the integrated fluorescence density (IntDen = sum of the grey scale values of each pixel * area of one pixel) expressed in arbitrary fluorescence units (AU). For cell clusters where it was challenging to demarcate the individual cell borders, the total F-actin density of the cluster was measured and averaged over the number of DAPI-stained nuclei in the cluster. All samples were imaged using consistent confocal camera settings for comparison.

### 2.8 Statistics

Data for this experiment were completed in triplicate, at least. Finalized measurements were analyzed as averages with standard deviations to represent error. A two-tailed, equal variance Student’s t-test was used to analyze single statistical comparisons. For multiple comparisons a two-way ANOVA was performed with a subsequent Tukey test. The Shapiro-Wilks test was used to assess normality, the Pearson correlation coefficient (r) was used to evaluate the linear correlation between two variables, and the t-distribution to evaluate the associated p-value. The correlative analysis involved comparing the average F-actin density per cell with bulk RNA expression levels. For all tests, a p-value of less than 0.05 was considered significant. Graph Pad Prism was used for all statistical analysis.

## 3 Results

### 3.1 Tunable microgels and their physical properties

**Figure 2:**
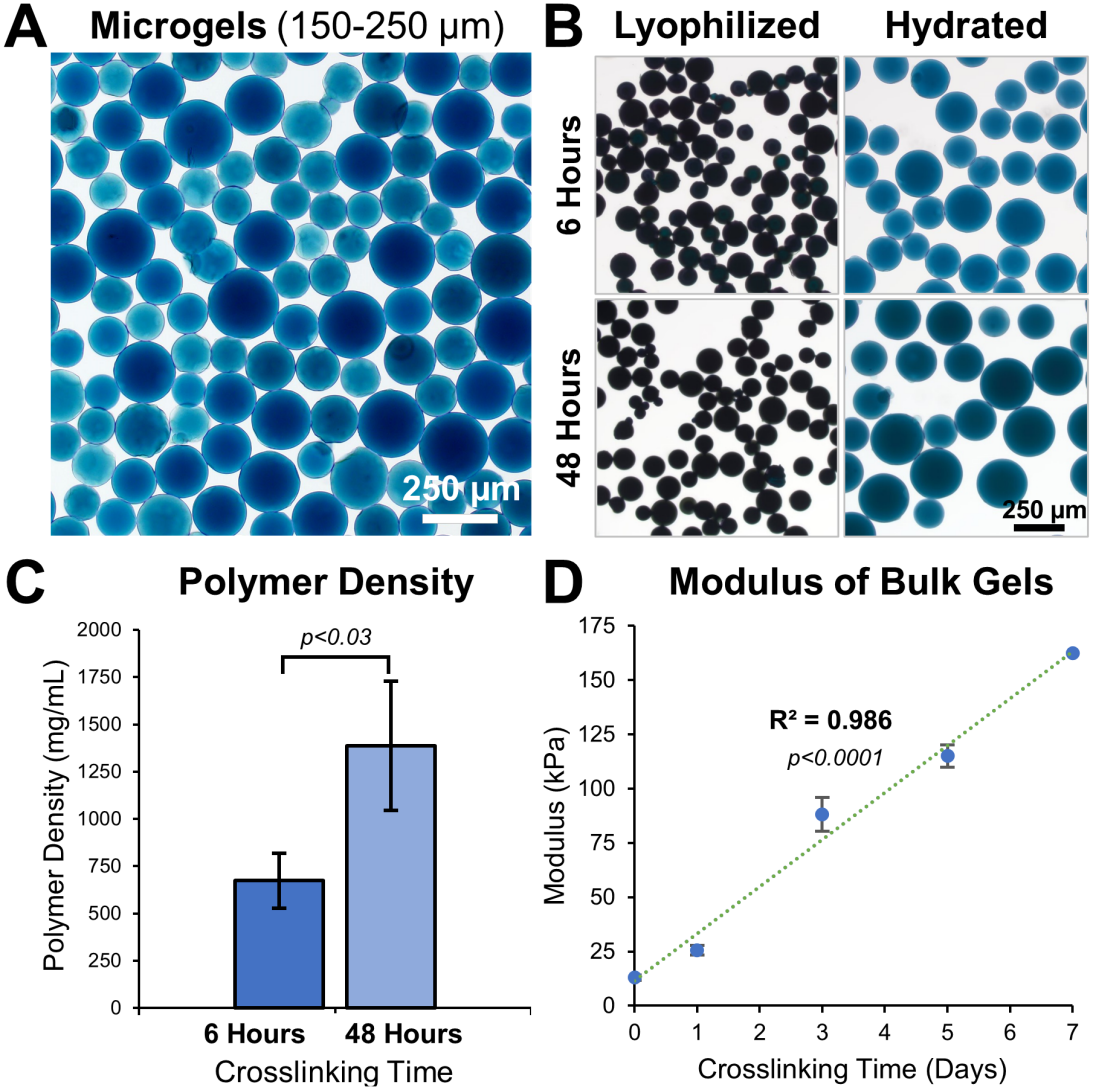
Tunable microgel fabrication and characterization. **(A)** Bright-field images of 48 hours crosslinked hydrated microgels of size range 150-250 μm. **(B)** Images of 6 hours and 48 hours crosslinked microgels before and after hydration in PBS. **(C)** Polymer density of 6 hours and 48 hours crosslinked microgels, n = 3 batches. **(D)** Young’s modulus of gelatin hydrogel disks show a linear increase with crosslinking time, n = 6.

Microgels were fabricated using a simple water-in-oil emulsification of solubilized gelatin followed by genipin crosslinking to the desirable extent. The size distribution of the microgels varied with the set speed of the propeller during the emulsification. Lower speeds (<500 rpm) generally resulted in microgels of a larger size range (150-400 µm), while higher speeds (>1000 rpm) resulted in smaller microgels (40-50 µm). To achieve narrow size ranges, nylon meshes were used to sort microgels to desired size ranges (**Figure 2A**, Hydrated 150-250 µm microgels). To achieve at least one-order-of-magnitude separation between the surface curvatures, we created microgels with two size ranges: 40-50 µm (κ = 0.045 µm-1) and 350-400 μm (κ = 0.005 µm^-1^). A mid-size range of 150-250 μm for material characterization purposes.

The material properties of a hydrogel can considerably influence cell behavior. By adjusting the crosslinking time, the microgel properties were easily fine-tuned (**Figure 2B**). Microgels crosslinked for 6 hours had a lower polymer density (674 ± 146 mg/mL) than microgels crosslinked for 48 hours (1386 ± 342 mg/mL, **Figure 2C**). Microgels with low crosslinking (6 hours) had a smaller volume-swelling ratio of 502 ± 68.7% and microgels with high crosslinking (48 hours) had a larger volume-swelling ratio of 855 ± 82.2% (**Figure 2B**).

We used bulk hydrogel samples crosslinked for different periods and performed compression testing to study their tunability. Solidified gelatin hydrogel disks, 5 mm high and 6 mm in diameter, were crosslinked for varying periods and subjected to compression tests. The elastic modulus of the disks ranged between 13.0 ± 1.22 kPa at day 0 and 163 ± 5.29 kPa at day 7 of crosslinking time, exhibiting a linear correlation (**Figure 2D**, R (2) = 0.986, p<0.0001, n=6).

### 3.2 Substrate curvature moderately influences macrophages attachment and spreading

**Figure 3:**
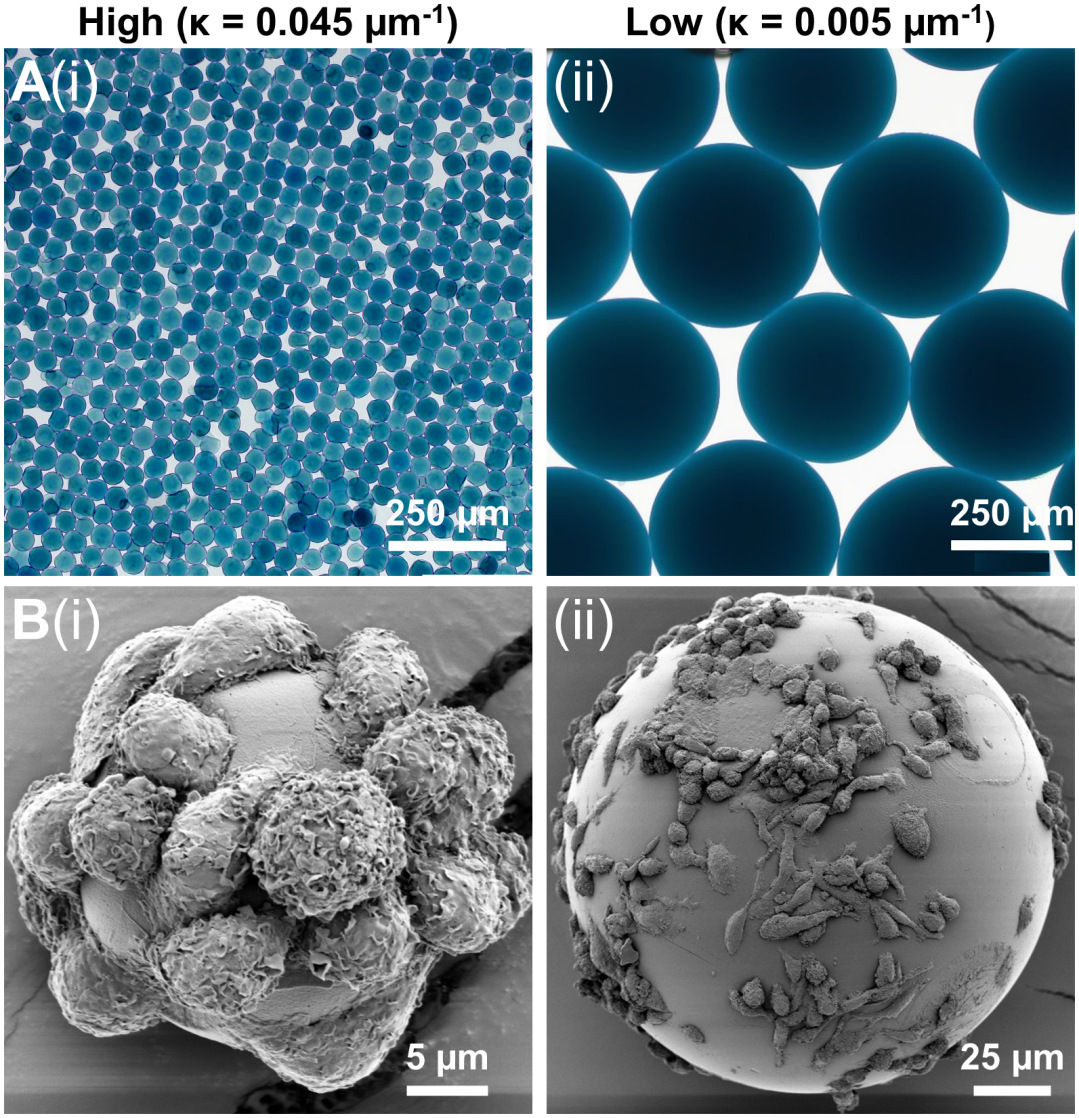
Macrophage attachment on low and high curvature microgels. **(A)** Bright-field microscopy images of the (i) high curvature (κ = 0.045 µm^-1^) and (ii) low curvature (κ = 0.005 µm^-1^) microgels showing uniform smooth surface morphology, **(B)** SEM images of macrophages seeded on (i) high curvature and (ii) low microgels showing morphological differences.

To investigate the effect of substrate curvature on macrophages cytoskeletal dynamics and phenotype, microgels with two degrees of curvature were selected: high curvature microgels with a diameter of 40-50 µm (κ = 0.045 µm^-1^, **Figure 3A**i) and lower curvature microgels with a diameter of 350-400 µm (κ = 0.005 µm^-1^, **Figure 3A**ii). These microgels were crosslinked for 48 hours to maintain a consistent modulus of around 50 kPa. After thorough washing with ethanol, followed by PBS and culture media, the microgels were seeded with macrophages in suspension cultures.

The macrophages quickly attached to the gelatin surface, covering both high and low curvature microgels within 24 hours. No significant difference in cell attachment was noticed between the high (**Figure 3B**i) and low (**Figure 3B**ii) curvature conditions. But the attached macrophages tended to be more elongated in the low curvature compared to the high curvature microgels (**Figure 3B**ii). On the high curvature microgels, macrophages tended to warp around the microgel, displaying a more compact morphology.

### 3.3 Substrate curvature modulate cytoskeletal dynamics and phenotype of macrophages

**Figure 4:**
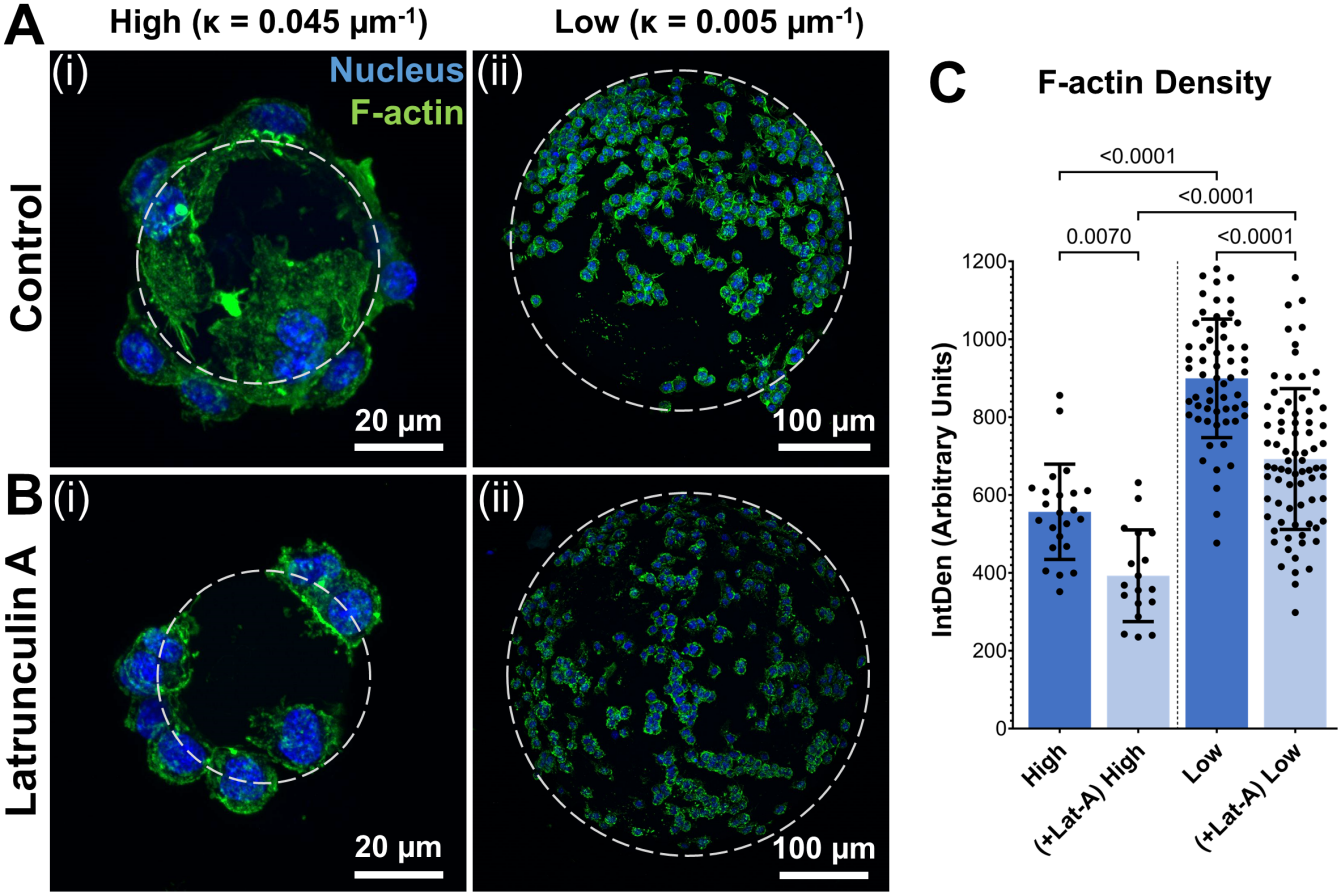
Substrate curvature modulates cytoskeletal dynamics. **(A)** Confocal stacks of Day 7 macrophages seeded on high (i) and low (ii) curvature microgels. **(B)** Confocal stacks of macrophages seeded on high (i) and low (ii) curvature microgels after latrunculin-A treatment for the last 24 hours of day 7 cultures. Green and blue fluorescence corresponds to cytoskeletal F-actin and nuclei, respectively. **(C)** F-actin density (IntDen) in macrophages seeded on microgels in the respective groups (n = 25) measured through ImageJ quantification (n>25 for each group).

To investigate the influence of substrate curvature on cytoskeletal dynamics and corresponding phenotypic changes, macrophages were cultured on low and high curvature microgels for 7 days and characterized through actin cytoskeleton staining and gene expression studies. Fluorescence staining and quantification of the cytoskeletal F-actin, on day 7, revealed that the macrophages on low curvature microgels exhibited a 64% higher F-actin density (898.87 ± 128.36 AU, **Figures 4A**i, **C**) compared to those on the high curvature microgels (548.70 ± 127.23 AU, *p<0.0001*, n=25, **Figures 4A**ii, **C**).

For further validation, selective inhibition of actin polymerization was performed using latrunculin-A (Lat-A) treatment for the last 24 hours of the 7-day cultures. Lat-A treatment significantly reduced F-actin density in both groups (F-actin density in Low = 725.12 ± 148.12 AU, *p<0.0001* and High = 404.69 ± 113.7 AU, *p<0.0001*) and led to a corresponding reduction in cell spreading in both groups (**Figures 4B, C**). Despite this reduction, the low curvature microgels maintained significantly (79%) higher F-actin density compared to high curvature microgels, indicating a strong influence on the cytoskeleton of macrophages.

Subsequently, we investigated the changes in immunophenotype of macrophages caused by alterations in actin polymerization. Actin polymerization is known to trigger the release of the transcription factor MRTF-A (24), which regulates expression levels of several genes involved in inflammatory responses. A panel of prominent inflammatory and anti-inflammatory genes, as well as genes implicated in actin cytoskeletal dynamics, was identified. The expression levels of these genes were quantified and compared after 1 week of culturing in low and high curvature microgels.

**Figure 5:**
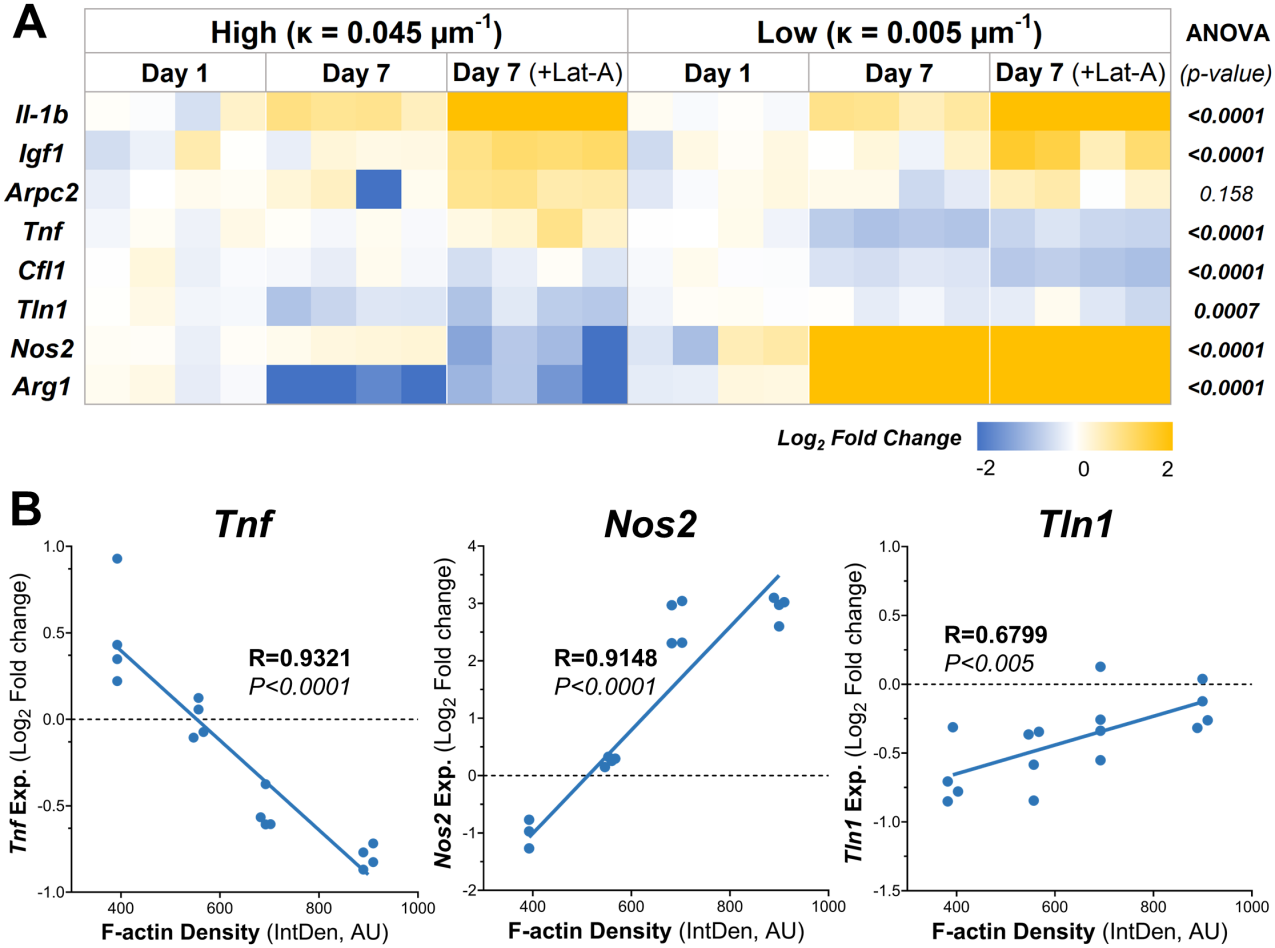
Substrate Curvature Modulate Macrophage Phenotype. **(A)** The relative gene expression levels of common inflammatory and anti-inflammatory markers in macrophages cultured on high and low curvature gels for 7 days, treated and untreated with Lat-A (n = 4). **(B)** Correlation plots of gene expression at day 7 with significant linear relationships with f-actin level. AU, Arbitrary units.

The relative expression levels of several key genes exhibited significant differences between macrophages cultures on low and high curvature microgels at day 7, both before and after Lat-A treatment (**Figure 5A**). Notably the pro-inflammatory gene *Tnf*, and the anti-inflammatory genes *Arg1* and *Nos2* showed robust curvature dependent expression patterns.

Low curvature condition led to a significant downregulation of *Tnf* (1.7-fold decrease*, p=0.0001*). Conversely, *Arg1* was significantly downregulated in high curvature conditions (4-fold, *p=0.0103*), while *Arg1 and Nos2 were* being significantly upregulated in low curvature conditions (7.7-fold, *p=0.0003*, and 7.6-fold *p<0.0001*, respectively). Interestingly, these trends persisted even after Lat-A treatment that significantly reduced the F-actin polymerization. Correlation analysis revealed a robust and significant linear correlation between F-actin levels and the expression of *Tnf* (R=0.9321, *p<0.0001*), *Arg1* (R=0.9148, *p<0.0001*), and *Tln1* (R=0.6799, *p<0.005*) (**Figure 5B**; **Tables 1**, **2**), indication a strong link between cytoskeletal dynamics and macrophage phenotype.

**Table 1:** Fold-change in the expression levels of prominent inflammatory genes.

*Gene*	High Curvature		Low Curvature	
	High κ Day 7	High κ Lat-A Day 7	Low κ Day 7	Low κ Lat-A Day 7
***Il1b***	0.81 ± 0.21	3.02 ± 0.24	0.77 ± 0.17	4.05 ± 0.13
***Igf1***	0.13 ± 0.24	1.10 ± 0.11	0.14 ± 0.27	1.20 ± 0.48
***Arpc2***	-0.34 ± 1.30	0.79 ± 0.14	-0.12 ± 0.35	0.39 ± 0.29
***Tnf***	0.0004 ± 0.19	0.48 ± 0.31	-0.80 ± 0.07	-0.54 ± 0.11
***Cfl1***	-0.09 ± 0.16	-0.24 ± 0.23	-0.39 ± 0.06	-0.79 ± 0.09
***Tln1***	-0.54 ± 0.23	-0.66 ± 0.24	-0.17 ± 0.16	-0.26 ± 0.28
***Nos2***	0.26 ± 0.08	-1.40 ± 0.82	2.92 ± 0.22	2.66 ± 0.40
***Arg1***	-2.06 ± 0.25	-1.42 ± 0.70	2.95 ± 0.18	3.76 ± 0.15

**Table 2:** Pairwise comparison of treatment conditions.

*Gene*	High Curvature			Low Curvature			High vs Low	
	Day 1 vs -Day 7	Day 1 vs Day 7 (+Lat-A)	Day 7 vs Day 7 (+Lat-A)	Day 1 vs -Day 7	Day 1 vs Day 7 (+Lat-A)	Day 7 vs Day 7 (+Lat-A)	Day 7	Day 7 (+Lat-A)
***Il1b***	**0.0006**	**<0.0001**	**<0.0001**	**0.001**	**<0.0001**	**<0.0001**	0.9999	**<0.0001**
***Igf1***	0.995	**0.0033**	**0.0099**	0.9908	**0.0014**	**0.0048**	>0.9999	0.9982
***Arpc2***	0.9729	0.5282	0.1307	0.9997	0.9327	0.8221	0.9946	0.9292
***Tnf***	>0.9999	**0.0087**	**0.0088**	**<0.0001**	**0.0032**	0.3088	**<0.0001**	**<0.0001**
***Cfl1***	0.9774	0.2664	0.6521	**0.0206**	**<0.0001**	**0.0171**	0.0857	**0.0009**
***Tln1***	**0.0178**	**0.0028**	0.9473	0.8574	0.5162	0.9887	0.1634	0.1018
***Nos2***	0.9763	**0.0103**	**0.0022**	**<0.0001**	**<0.0001**	0.9728	**<0.0001**	**<0.0001**
***Arg1***	**<0.0001**	**0.0003**	0.1491	**<0.0001**	**<0.0001**	**0.0409**	**<0.0001**	**<0.0001**

Values in bold represent statistically significant p-values (< 0.05).

## 4 Discussion

Inflammation is a critical and tightly regulated aspect of the immune response, and its dysregulation often leads to debilitating diseases (1–3). While small molecules, such as NSAIDs (18), have effectiveness in modulating inflammatory responses, they can cause significant off-target effects, such as gastrointestinal complications (27) and systemic issues. This underscores the need for alternative therapeutic approaches that do not rely solely on biochemical interventions.

It is well recognized that mechanical forces play a vital role in regulating major cellular events, including inflammatory response, tissue development, regeneration, and healing after trauma (28). However, the majority of the work has focused on inflammation mediated by biochemical signals, with less attention given to the physical parameters of the inflammatory environment, especially matrix mechanics. These physical parameters, particularly matrix stiffness and curvature, are crucial in modulating immunophenotypes and potentially key to developing the next generation of therapies.

In this study, we address this gap by investigating how substrate curvature influences macrophage behavior and immunophenotype. Macrophages are innate immune cells known for their significant influence on the inflammatory response. They exhibit remarkable plasticity in response to both biochemical and biophysical cues. This plasticity allows them to both initiate and resolve inflammation, making them ideal candidates for therapeutic manipulation. By elucidating the impact of physical properties like curvature on macrophage activation, we open new avenues for therapeutic interventions that minimize off-target effects and harness the potential of biophysical cues to regulate inflammation. This approach could lead to more precise and effective treatments for inflammatory diseases.

### 4.1 A simple and tunable microgel system

To investigate the impact of surface curvature on cellular behavior, we developed a microgel system using gelatin—a well-characterized biomaterial crosslinked with the biocompatible agent genipin—to create microspheres in various sizes. Gelatin, a product of partial collagen hydrolysis, preserves key bioactive motifs, such as RGD and GFOGER sequences, that support cellular adhesion. Its naturally occurring integrin-binding sites were essential to this system, allowing for cellular attachment without further chemical modification. Notably, gelatin-binding integrins, particularly α5β1 and αvβ3, are expressed in multiple cell types, including macrophages, and recognize these bioactive sequences effectively (29). Genipin was selected as the crosslinking agent due to its specificity for lysine residues, forming stable links via amine groups while preserving the functionality of RGD sequences. This specificity provides a significant advantage over carbodiimide-based chemistries, which can disrupt essential motifs by targeting carboxylate groups on glutamate and aspartate residues.

The stiffness and polymer density of the microgels can be precisely tuned by adjusting the crosslinking time, and all microgels used in this study were crosslinked for 48 hours to ensure consistent material properties across experiments. Previously, using atomic force microscopy (AFM) we showed that fully crosslinked microgels had a stiffness of 189 kPa (33), comparable to fully crosslinked bulk gelatin hydrogels, which require significantly longer crosslinking times. Scanning electron microscopy (SEM) and focused ion beam slicing confirmed the tight, densely bound structure of the gelatin material (33).

The size—and thus the curvature—of the microgels was carefully controlled by sorting them into defined size ranges, enabling systematic investigations of curvature-dependent cellular responses. Importantly, the microgels are fully degradable, allowing integration with native extracellular matrix (ECM) components while retaining their structural integrity for several weeks. This degradability, coupled with their tunable properties, makes them highly suitable for both high-throughput experimental studies and therapeutic applications (30, 31).

### 4.2 Curvature-sensing and cytoskeletal dynamics

Cells sense substrate curvature primarily through alterations in stress fiber (SF) formation and cytoskeletal tension. On flat surfaces, cells readily form SFs to generate the tension required for motility. In contrast, on curved surfaces, SF formation is energetically costly due to the bending required, leading to reduced SF assembly and a corresponding decrease in actin polymerization. This reduction in SF formation influences the mechanical forces that cells experience, which in turn modulates cellular behavior.

In this study, the RGD-rich surface of gelatin microgels provided a robust platform for macrophage adhesion, enabling an investigation into how substrate curvature influences cellular morphology and behavior. Once adhered, macrophages exhibited pronounced curvature-dependent differences in shape and phenotype (**Figures 4**, **5**). These distinctions likely stem from the varying bending energy required for cells to conform to substrates of different curvatures, a process mediated by integrin-RGD interactions (34). Similar sensitivity to substrate curvature has been reported in mesenchymal stromal cells (MSCs) and fibroblasts, highlighting a conserved mechanobiological response across cell types (32–34).

Typically, cells avoid adhering to high curvature surfaces due to the increased energetic costs associated with bending and wrapping. However, the uniformly curved surface of spheres forces cells to adapt, expending the necessary bending energy to establish adhesion, which may have phenotypic consequences. In macrophages, this adhesion process is mediated by integrins α5β1 and αvβ3, which specifically bind to RGD motifs on the microgel surface (29). While the downstream signaling cascades activated by these interactions remain to be fully elucidated, they likely involve cytoskeletal remodeling processes that will be the focus of future investigations.

Our findings align with previous studies demonstrating that cells spread more extensively on low-curvature surfaces while exhibiting restricted spreading on high-curvature substrates. In the case of the low-curvature microgels, reduced bending energy requirements facilitated efficient macrophage spreading and elevated F-actin polymerization. These results suggest that curvature modulates cytoskeletal dynamics by influencing the energetic landscape of cell adhesion and spreading, providing insights into how biophysical cues shape cellular responses.

### 4.3 F-actin polymerization and transcriptomic modulation in macrophages

Macrophages employ several mechanosensory pathways that activate downstream signaling cascades and transcription factors, including YAP/TAZ (Yes-Associated Protein and Transcriptional Coactivator with PDZ-Binding Motif), NF-κB (Nuclear Factor-κB), AP-1 (Activator Protein-1), SMADs, STATs (Signal Transducer and Activator of Transcription), IRFs (Interferon Regulatory Factors), and β-catenin. For this study, we focused on the myocardin-related transcription factor A (MRTF-A) pathway, which plays a pivotal role in macrophage gene regulation and exhibits a well-characterized mechanosensitive response to actin cytoskeletal dynamics.

F-actin polymerization is driven by the release of monomeric G-actin, which unbinds from MRTF-A, allowing MRTF-A to translocate to the nucleus (**Figure 1**). Once nuclear, MRTF-A associates with serum response factor (SRF) to activate the transcription of specific genes, including key inflammatory mediators such as *Tnf*, *Nos2*, and *Il6 (* 35, 36). We hypothesized that actin-dependent transcriptional changes modulate macrophage phenotype in response to substrate curvature. To test this, we cultured macrophages on high- and low-curvature microgels and assessed curvature-induced differences in inflammatory and cytoskeletal gene expression.

Our results showed that macrophages on low-curvature microgels exhibited higher F-actin levels compared to those on high-curvature microgels after one week of culturing. Latrunculin A (LatA), which inhibits F-actin polymerization, proportionally reduced F-actin levels in both groups. This allowed us to assess the influence of F-actin on macrophage phenotype at differential F-actin levels and presumably the subsequent MRTF-A nuclear translocation as well. Two inflammatory genes exhibited strong linear relationships with F-actin levels: *Tnfα* showed a strong negative correlation, while *Nos2* showed a strong positive correlation. *Tnfα* is a major component of the innate inflammatory response, initiating the inflammatory cascade by promoting the expression of multiple pro-inflammatory proteins (37). *Nos2* exhibits a pro-inflammatory effect at low concentrations, while at higher concentrations it can mount an anti-inflammatory effect by promoting the apoptosis of inflammatory cells (38). Additionally, the cytoskeletal gene, *Tln1* (talin-1), essential for linking activated integrins to cytoskeletal actin and focal adhesion assembly (39), shows a strong positive correlation with F-actin levels.

These results highlight the role of substrate curvature in modulating F-actin dynamics and MRTF-A-mediated transcription. While macrophages cultured on high-curvature substrates showed gene expression changes consistent with an anti-inflammatory bias, further studies are needed to confirm a definitive phenotypic shift toward the M2 spectrum. By fine-tuning the physical properties of the microgels, such as curvature, it may be possible to strategically direct macrophage responses to achieve desired inflammatory or reparative outcomes. These findings provide a compelling foundation for the development of biophysically guided therapeutic strategies aimed at controlling inflammation and enhancing tissue regeneration.

## 5 Summary and conclusion

This study demonstrates the utility of our microgel system as a versatile platform to investigate the effects of physical characteristics on the phenotypes of adherent cells. The tunable properties of the microgels enable the evaluation of a broad range of material attributes, either independently or in combination, providing a robust tool for mechanobiological research.

Our investigation into the impact of curvature on macrophage phenotype revealed that F-actin dynamics play a crucial role in modulating the production of inflammatory mediators. These findings offer valuable insights into how physical cues, such as curvature, can influence cell behavior and phenotype. This knowledge has significant implications for the design of biomaterials, suggesting that shape and other physical properties alone can be harnessed to enhance therapeutic outcomes.

By leveraging the interplay between physical cues and cellular responses, biomaterials can be tailored to modulate the cell secretome and reduce the off-target effects often associated with chemical mediators. Overall, this study underscores the importance of structural and material properties in biomaterial design and highlights their potential to drive innovative therapeutic strategies.

## Data Availability

The raw data supporting the conclusions of this article will be made available by the authors, without undue reservation.

## References

[1] Pahwa R Goyal A Bansal P Jialal I. Chronic Inflammation. In: StatPearls. Treasure Island (FL): StatPearls Publishing. (2023).

[2] Chen L Deng H Cui H Fang J Zuo Z Deng J. Inflammatory responses and inflammation-associated diseases in organs. Oncotarget. (2018) 9:7204. doi: 10.18632/oncotarget.23208 29467962 PMC5805548

[3] Furman D Campisi J Verdin E Carrera-Bastos P Targ S Franceschi C. Chronic inflammation in the etiology of disease across the life span. Nat Med. (2019) 25:1822–32. doi: 10.1038/s41591-019-0675-0 PMC7147972 31806905

[4] Gauthier T Chen W. Modulation of macrophage immunometabolism: A new approach to fight infections. Front Immunol. (2022) 13:780839. doi: 10.3389/fimmu.2022.780839 35154105 PMC8825490

[5] Spiller KL Freytes DO Vunjak-Novakovic G. Macrophages modulate engineered human tissues for enhanced vascularization and healing. Ann Biomed Eng. (2015) 43:616–27. doi: 10.1007/s10439-014-1156-8 PMC4380684 25331098

[6] Dong L Wang C. Harnessing the power of macrophages/monocytes for enhanced bone tissue engineering. Trends Biotechnol. (2013) 31:342–6. doi: 10.1016/j.tibtech.2013.04.001 23623371

[7] Brown BN Ratner BD Goodman SB Amar S Badylak SF. Macrophage polarization: an opportunity for improved outcomes in biomaterials and regenerative medicine. Biomaterials. (2012) 33:3792–802. doi: 10.1016/j.biomaterials.2012.02.034 PMC3727238 22386919

[8] Murray PJ. Macrophage polarization. Annu Rev Physiol. (2017) 79:541–66. doi: 10.1146/annurev-physiol-022516-034339 27813830

[9] Wang S Liu Y Wang X Chen L Huang W Xiong T. Modulating macrophage phenotype for accelerated wound healing with chlorogenic acid-loaded nanocomposite hydrogel. J Controlled Release. (2024) 369:420–43. doi: 10.1016/j.jconrel.2024.03.054 38575075

[10] Ye J Xie C Wang C Huang J Yin Z Heng BC. Promoting musculoskeletal system soft tissue regeneration by biomaterial-mediated modulation of macrophage polarization. Bioactive Mater. (2021) 6:4096–109. doi: 10.1016/j.bioactmat.2021.04.017 PMC8091177 33997496

[11] Scheraga RG Abraham S Niese KA Southern BD Grove LM Hite RD. TRPV4 mechanosensitive ion channel regulates lipopolysaccharide-stimulated macrophage phagocytosis. J Immunol. (2016) 196:428–36. doi: 10.4049/jimmunol.1501688 PMC4684994 26597012

[12] McWhorter FY Wang T Nguyen P Chung T Liu WF. Modulation of macrophage phenotype by cell shape. Proc Natl Acad Sci. (2013) 110:17253–8. doi: 10.1073/pnas.1308887110 PMC3808615 24101477

[13] Baranov MV Kumar M Sacanna S Thutupalli S Van den Bogaart G. Modulation of immune responses by particle size and shape. Front Immunol. (2021) 11:607945. doi: 10.3389/fimmu.2020.607945 33679696 PMC7927956

[14] Schwartz DM Kanno Y Villarino A Ward M Gadina M O'Shea JJ. JAK inhibition as a therapeutic strategy for immune and inflammatory diseases. Nat Rev Drug Discovery. (2017) 16:843–62. doi: 10.1038/nrd.2017.201 29104284

[15] Mountziaris PM Mikos AG. Modulation of the inflammatory response for enhanced bone tissue regeneration. Tissue Eng Part B: Rev. (2008) 14:179–86. doi: 10.1089/ten.teb.2008.0038 PMC2962857 18544015

[16] Hu Z Ma C Rong X Zou S Liu X. Immunomodulatory ECM-like microspheres for accelerated bone regeneration in diabetes mellitus. ACS Appl materials interf. (2018) 10:2377–90. doi: 10.1021/acsami.7b18458 PMC6437671 29280610

[17] Choy EH De Benedetti F Takeuchi T Hashizume M John MR Kishimoto T. Translating IL-6 biology into effective treatments. Nat Rev Rheumatol. (2020) 16:335–45. doi: 10.1038/s41584-020-0419-z PMC7178926 32327746

[18] Haley RM von Recum HA. Localized and targeted delivery of NSAIDs for treatment of inflammation: A review. Exp Biol Med. (2019) 244:433–44. doi: 10.1177/1535370218787770 PMC6546999 29996674

[19] Irwin EF Saha K Rosenbluth M Gamble LJ Castner DG Healy KE. Modulus-dependent macrophage adhesion and behavior. J Biomater Sci Polymer Edition. (2008) 19:1363–82. doi: 10.1163/156856208786052407 18854128

[20] Kalashnikov N Moraes C. Substrate viscoelasticity affects human macrophage morphology and phagocytosis. Soft Matter. (2023) 19:2438–45. doi: 10.1039/D2SM01683D 36930245

[21] Luu TU Liu WF. Regulation of macrophages by extracellular matrix composition and adhesion geometry. Regen Eng Trans Med. (2018) 4:238–46. doi: 10.1007/s40883-018-0065-z

[22] Luu TU Gott SC Woo BW Rao MP Liu WF. Micro-and nanopatterned topographical cues for regulating macrophage cell shape and phenotype. ACS Appl materials interf. (2015) 7:28665–72. doi: 10.1021/acsami.5b10589 PMC4797644 26605491

[23] Bade ND Kamien RD Assoian RK Stebe KJ. Curvature and Rho activation differentially control the alignment of cells and stress fibers. Sci Adv. (2017) 3:e1700150. doi: 10.1126/sciadv.1700150 28913421 PMC5587136

[24] Olson EN Nordheim A. Linking actin dynamics and gene transcription to drive cellular motile functions. Nat Rev Mol Cell Biol. (2010) 11:353–65. doi: 10.1038/nrm2890 PMC3073350 20414257

[25] An R. MRTF may be the missing link in a multiscale mechanobiology approach toward macrophage dysfunction in space. Front Cell Dev Biol. (2022) 10:997365. doi: 10.3389/fcell.2022.997365 36172272 PMC9510870

[26] Park E Hart ML Rolauffs B Stegemann JP T. Annamalai R. Bioresponsive microspheres for on-demand delivery of anti-inflammatory cytokines for articular cartilage repair. J Biomed Mater Res Part A. (2020) 108:722–33. doi: 10.1002/jbm.a.v108.3 PMC6995991 31788947

[27] Badri W Miladi K Agha Nazari Q Greige-Gerges H Fessi H Elaissari AE. Encapsulation of NSAIDs for inflammation management: overview, progress, challenges and prospects. Int J Pharma. (2016) 515:757–73. doi: 10.1016/j.ijpharm.2016.11.002 27829170

[28] Huse M. Mechanical forces in the immune system. Nat Rev Immunol. (2017) 17:679–90. doi: 10.1038/nri.2017.74 PMC6312705 28757604

[29] Davidenko N Schuster CF Bax DV Farndale RW Hamaia S Best SM. Evaluation of cell binding to collagen and gelatin: a study of the effect of 2D and 3D architecture and surface chemistry. J materials sci Mater Med. (2016) 27:148. doi: 10.1007/s10856-016-5763-9 PMC5007264 27582068

[30] Patrick MD Annamalai RT. Licensing microgels prolong the immunomodulatory phenotype of mesenchymal stromal cells. Front Immunol. (2022) 13. doi: 10.3389/fimmu.2022.987032 PMC9433901 36059508

[31] Patrick MD Keys JF Suresh Kumar H Annamalai RT. Injectable nanoporous microgels generate vascularized constructs and support bone regeneration in critical-sized defects. Sci Rep. (2022) 12:1–18. doi: 10.1038/s41598-022-19968-x 36138042 PMC9499928

[32] Lee SJ Yang S. Substrate curvature restricts spreading and induces differentiation of human mesenchymal stem cells. Biotechnol J. (2017) 12:1700360. doi: 10.1002/biot.201700360 28731631

[33] Callens SJ Uyttendaele RJ Fratila-Apachitei LE Zadpoor AA. Substrate curvature as a cue to guide spatiotemporal cell and tissue organization. Biomaterials. (2020) 232:119739. doi: 10.1016/j.biomaterials.2019.119739 31911284

[34] Lee SJ Yang S. Micro glass ball embedded gels to study cell mechanobiological responses to substrate curvatures. Rev Sci Instrum. (2012) 83:094302. doi: 10.1063/1.4751869 23020396

[35] Selvaraj A Prywes R. Expression profiling of serum inducible genes identifies a subset of SRF target genes that are MKL dependent. BMC Mol Biol. (2004) 5:1–15. doi: 10.1186/1471-2199-5-13 15329155 PMC516031

[36] Jain N Vogel V. Spatial confinement downsizes the inflammatory response of macrophages. Nat materials. (2018) 17:1134–44. doi: 10.1038/s41563-018-0190-6 PMC6615903 30349032

[37] Bradley J. TNF-mediated inflammatory disease. J Pathol. (2008) 214:149–60. doi: 10.1002/path.v214:2 18161752

[38] Coleman JW. Nitric oxide in immunity and inflammation. Int Immunopharmacol. (2001) 1:1397–406. doi: 10.1016/S1567-5769(01)00086-8 11515807

[39] Calderwood DA Campbell ID Critchley DR. Talins and kindlins: partners in integrin-mediated adhesion. Nat Rev Mol Cell Biol. (2013) 14:503–17. doi: 10.1038/nrm3624 PMC4116690 23860236

